# Perspectives of Patients About Immediate Access to Test Results Through an Online Patient Portal

**DOI:** 10.1001/jamanetworkopen.2023.3572

**Published:** 2023-03-20

**Authors:** Bryan D. Steitz, Robert W. Turer, Chen-Tan Lin, Scott MacDonald, Liz Salmi, Adam Wright, Christoph U. Lehmann, Karen Langford, Samuel A. McDonald, Thomas J. Reese, Paul Sternberg, Qingxia Chen, S. Trent Rosenbloom, Catherine M. DesRoches

**Affiliations:** 1 Department of Biomedical Informatics, Vanderbilt University Medical Center, Nashville, Tennessee; 2 Department of Emergency Medicine, UT Southwestern Medical Center, Dallas, Texas; 3 Clinical Informatics Center, UT Southwestern Medical Center, Dallas, Texas; 4 Department of Medicine, University of Colorado Anschutz Medical Campus, Aurora; 5 Department of Clinical Informatics, University of California Davis Health, Sacramento; 6 Department of Medicine, Beth Israel Deaconess Medical Center, Boston, Massachusetts; 7 Department of Medicine, Harvard Medical School, Boston, Massachusetts; 8 Department of Pediatrics, UT Southwestern Medical Center, Dallas, Texas; 9 Department of Insights and Operations, Vanderbilt University Medical Center, Nashville, Tennessee; 10 Department of Ophthalmology and Visual Sciences, Vanderbilt University Medical Center, Nashville, Tennessee; 11 Department of Biostatistics, Vanderbilt University Medical Center, Nashville, Tennessee; 12 Department of Medicine, Vanderbilt University Medical Center, Nashville, Tennessee; 13 Department of Pediatrics, Vanderbilt University Medical Center, Nashville, Tennessee

## Abstract

**Importance:**

The 21st Century Cures Act Final Rule mandates the immediate electronic availability of test results to patients, likely empowering them to better manage their health. Concerns remain about unintended effects of releasing abnormal test results to patients.

**Objective:**

To assess patient and caregiver attitudes and preferences related to receiving immediately released test results through an online patient portal.

**Design, Setting, and Participants:**

This large, multisite survey study was conducted at 4 geographically distributed academic medical centers in the US using an instrument adapted from validated surveys. The survey was delivered in May 2022 to adult patients and care partners who had accessed test results via an online patient portal account between April 5, 2021, and April 4, 2022.

**Exposures:**

Access to test results via a patient portal between April 5, 2021, and April 4, 2022.

**Main Outcomes and Measures:**

Responses to questions related to demographics, test type and result, reaction to result, notification experience and future preferences, and effect on health and well-being were aggregated. To evaluate characteristics associated with patient worry, logistic regression and pooled random-effects models were used to assess level of worry as a function of whether test results were perceived by patients as normal or not normal and whether patients were precounseled.

**Results:**

Of 43 380 surveys delivered, there were 8139 respondents (18.8%). Most respondents were female (5129 [63.0%]) and spoke English as their primary language (7690 [94.5%]). The median age was 64 years (IQR, 50-72 years). Most respondents (7520 of 7859 [95.7%]), including 2337 of 2453 individuals (95.3%) who received nonnormal results, preferred to immediately receive test results through the portal. Few respondents (411 of 5473 [7.5%]) reported that reviewing results before they were contacted by a health care practitioner increased worry, though increased worry was more common among respondents who received abnormal results (403 of 2442 [16.5%]) than those whose results were normal (294 of 5918 [5.0%]). The result of the pooled model for worry as a function of test result normality was statistically significant (odds ratio [OR], 2.71; 99% CI, 1.96-3.74), suggesting an association between worry and nonnormal results. The result of the pooled model evaluating the association between worry and precounseling was not significant (OR, 0.70; 99% CI, 0.31-1.59).

**Conclusions and Relevance:**

In this multisite survey study of patient attitudes and preferences toward receiving immediately released test results via a patient portal, most respondents preferred to receive test results via the patient portal despite viewing results prior to discussion with a health care professional. This preference persisted among patients with nonnormal results.

## Key Points

**Question:**

What are patient attitudes and perspectives related to viewing immediately released test results through an online patient portal?

**Findings:**

In this survey study of 8139 respondents at 4 US academic medical centers, 96% of patients preferred receiving immediately released test results online even if their health care practitioner had not yet reviewed the result. A subset of respondents experienced increased worry after receiving abnormal results.

**Meaning:**

In this study, most patients supported receiving immediately released test results via a patient portal, but some patients experienced increased worry, especially when test results were abnormal.

## Teaser

This survey study examines respondents’ attitudes and perspectives about receiving immediately released medical test results through an online patient portal.

## Introduction

The US Office of the National Coordinator for Health Information Technology’s Final Rule implementing the information-blocking portion of the 21st Century Cures Act went into effect on April 5, 2021. The Final Rule mandates the immediate electronic availability of nearly all test results, medication lists, and clinical notes to patients and care partners upon their request.^1^ Improved access to personal health information allows patients to manage their health care and supports coordination efforts among patients, care partners, and health care teams.^2,3,4^ However, the benefit to immediate release of test results may be offset by unintended consequences to patient well-being and confidentiality.^5,6^

Online patient portals have emerged as important tools for facilitating engagement and enabling patients to access health information from their medical records, review educational resources, participate in medical decision-making, and communicate with clinicians.^7,8,9^ Prior to the Cures Act, individual health systems could choose which health information to share via portals. Many health systems shared laboratory and imaging results; some also shared clinical notes.^10,11,12^ However, many health systems suppressed or delayed the release of certain results, collectively defined as information blocking. Information blocking was intended to provide health care practitioners time to review and discuss results with patients when indicated. Delays and suppression were common for results associated with misinterpretation or emotional distress (eg, HIV testing, genetic testing for Huntington disease, or tissue biopsy results concerning for malignant tumors).^13^ Early research suggested that immediate release of test results was associated with more patients viewing their health data. A study^14^ since the Final Rule went into effect showed a 4-fold increase in the number of results viewed by patients prior to clinician counseling and a doubling of the number of patient-initiated messages sent to clinicians within 6 hours of viewing results.

Full access to medical records has been advocated as a strategy for strengthening patient-clinician relationships.^15,16,17^ Most patients want unrestricted access to their medical records.^18,19^ The OpenNotes collaborative established the immediate release of clinical notes (ie, open notes) as best practice.^11,20,21,22^ However, the practice of immediately releasing test results without context provided by clinician counseling (ie, open results) remains controversial.^5^ While portal users may be satisfied receiving test results online, portals provide inadequate guidance on how to interpret sensitive or abnormal results, which may contribute to negative emotions.^5,23^

Some patients and clinicians prefer to discuss sensitive or abnormal results synchronously to review results, answer questions, and formulate a treatment plan.^24^ Pilot studies suggest varied patient preferences about how and when to receive results.^23,25^ Result release strategies should align with patient preferences and minimize distress. To best design release strategies, we first must understand patient attitudes and preferences related to open results, which have not been widely studied. To address this gap, we surveyed a large cohort of patients and care partners receiving immediately released test results via a patient portal at 4 geographically diverse academic medical centers.

## Methods

### Study Setting and Participants

This survey study was fielded at 4 US academic medical centers serving diverse geographic regions, including the Pacific West (University of California, Davis Health [UC Davis Health]), Rocky Mountain Region (University of Colorado Anschutz Medical Center [CU Anschutz]), Southwest (University of Texas Southwestern Medical Center [UTSW]), and Southeast (Vanderbilt University Medical Center [VUMC]). Eligible participants included English-speaking adult patients and their designated caregivers with email addresses documented in the electronic health record (EHR) who accessed test results via a patient portal in the calendar year after the implementation of the Cures Act (April 5, 2021, to April 4, 2022). UTSW, UC Davis Health, and VUMC recruited participants from registries of patients who had previously consented to be contacted for research.^26^ CU Anschutz did not have a comparable registry and instead invited all eligible patients who had viewed results on the portal in the month preceding the study. All survey sites use the Epic EHR and MyChart patient portal (Epic Systems Corporation). The institutional review board at each site approved all study procedures and granted waivers of informed consent since patient identifiers were not collected. We followed the American Association for Public Opinion Research (AAPOR) reporting guideline.

### Survey Instrument

We adapted a previously validated instrument designed to evaluate patient perceptions about open notes^11,27^ and later about the immediate release of COVID-19 test results.^23^ We piloted this instrument with VUMC’s Patient Advisory Council.^25^ The instrument included 29 questions in 6 domains: (1) demographics and portal user role, (2) test result information, (3) result review behaviors, (4) education and health care practitioner follow-up, (5) effects on health and well-being, and (6) preferences for future results. Respondents who reviewed multiple test results during the study period could select multiple result types in their response.

We implemented the survey using REDCap.^28^ The instrument is available in the eAppendix in Supplement 1. We built the first REDCap project at VUMC and replicated it at the other sites using REDCap’s sharing tools. This facilitated identical content except for site-specific branding.

### Survey Procedure

In May 2022, we emailed eligible participants an explanation of the study and a survey link. Participants who did not initially complete the survey received 2 follow-up emails sent approximately 10 days apart. The survey remained open for 33 days. Each site managed local survey distribution and data collection. Participants were not compensated.

### Statistical Analysis

We calculated descriptive statistics from each site for all survey questions. Question-level data are reported as the count and percentage of responses among available participants for each respective question. We also computed descriptive statistics, stratifying by whether patients were precounseled (eTable in Supplement 1).

We then evaluated participant-reported level of worry as a function of whether participants perceived test results as normal or not normal and whether they were precounseled (ie, the reason for the test was explained before testing). Worry was represented as an ordinal categorical variable with the following values: (1) “I was never worried,” (2) “much less worried,” (3) “less worried,” (4) “no change,” (5) “more worried,” and (6) “much more worried.” The independent variables (test result normality and precounseling) were represented as dichotomous variables. The not normal category was an aggregation of responses of “not normal,” “other,” and “unknown” on the survey question.

We plotted the all-site proportion of participants with each level of worry, stratified by normal vs not normal test results. We then performed a prospective meta-analysis using random-effects models to pool site-specific odds of worry as a function of test result normality and precounseling. Site-specific odds were calculated using multivariable proportional odds ordinal logistic regression models evaluating worry as a function of test result normality and whether participants were precounseled. For random-effects models, we used restricted maximum likelihood estimation for model generation and Hartung-Knapp-Sidik-Jonkman–style test statistics.^29^ Site-specific models were adjusted by identical covariates to account for potential confounding. We selected candidate covariates based on clinical expertise and known patient portal disparities. We collapsed covariates representing less than 2% of the study population into “other” categories. We required all sites to use identical models to facilitate meta-analysis via the random-effects model; thus, covariates with insufficient samples at any of the sites were removed from all 4 sites’ models. We evaluated for collinearity using Spearman correlation coefficients. No covariate pairs had correlation coefficients greater than 0.5, so we did not remove any covariates due to collinearity. Finally, variables were removed for missingness over 30%. We performed random forest imputation using all candidate variables (including those omitted due to missingness).^30,31^ The selection process yielded the following covariates: age, comorbidity count, employment status, health care worker status, ethnicity, race, language, test type, precounseling, and mode of contact regarding test results. Race and ethnicity were assessed by participant self-report. Ethnicity categories were Spanish or Latino, and race categories were American Indian or Pacific Native, Asian, Black or African American, Native Hawaiian or Pacific Islander, White, and other (listed as an option on the survey). Continuous variables (comorbidity count and age) were modeled as restricted cubic splines with 3 knots. Restricted cubic splines are piecewise polynomials that allow models to account for nonlinear relationships and are restricted to linear functions in the heads and tails to avoid erratic behavior at the extremes.^32^ Before model fitting, we examined the proportional odds assumption using univariate models plotted across outcomes strata using the mean value of each variable per strata.^33^

We also fit site-specific multivariable models including first- and second-order interaction terms to test for interactions between test result normality and precounseling. This was performed to assess a potential association between test result normality and precounseling, which might require the effects of each to be modeled differently given the status of the other. Pooled odds ratios (ORs) for worry as a function of test result normality and precounseling are reported along with *I*^2^ statistics for heterogeneity.^34^ We defined *I*^2^ less than 25% as low heterogeneity, between 25% and 50% as moderate heterogeneity, and greater than 50% as high heterogeneity.^35^ Analyses were performed using R, version 4.1.2 (R Project for Statistical Computing) with the rms package for single-site regression and the metafor package for meta-analysis.^32,36^ We set 1-sided *P* < .05 for likelihood ratio χ^2^ testing.

## Results

Of 43 380 surveys delivered, there were 8139 participants (18.8%), of whom 5129 (63.0%) identified as female, 2895 (35.6%) as male, and 115 (1.4%) as other or unknown gender. A total of 120 (1.5%) were American Indian or Pacific Native; 250 (3.1%), Asian; 428 (5.3%), Black or African American; 23 (0.2%), Native Hawaiian or Pacific Islander; 6900 (84.8%), White; and 245 (3.0%) other race; 420 (5.2%) were Spanish or Latino. Most patients spoke English as their primary language (7690 [94.5%]). The median age of participants was 64 years (IQR, 50-72 years). Table 1 provides detailed respondent demographic characteristics. A total of 6306 of 7856 respondents (80.3%) reported reviewing at least 1 test result in the past month, and 5767 of 6245 (92.3%) reported receiving precounseling. Most tests were blood tests (4730 of 6276 [75.4%]). Imaging or biopsies accounted for 3044 of 6276 tests (48.5%). Most respondents reported normal findings (3582 of 6246 [57.3%]) (Table 2). Among 6200 respondents who reviewed results, 5418 (87.4%) reported being contacted by a health care practitioner about the result. Most commonly, communication occurred through a patient portal message (3783 of 6200 [61.0%]), during a clinic or telemedicine visit (1157 of 6200 [18.7%]), or through a telephone call (1108 of 6200 [17.9%]). Of 5318 patients who sought additional information after reviewing their results, 2123 (39.9%) conducted an internet search. When asked about their preferences for contacts about future test results, 7046 of 7814 respondents (90.2%) indicated that they would prefer result delivery via the patient portal. Nearly all respondents (7520 of 7859 [95.7%]) indicated that they wanted to receive results through the patient portal as soon as results were available, even if their health care practitioner had not yet reviewed a result. Furthermore, 2337 of 2453 respondents (95.3%) who received not normal test results similarly indicated that they wanted to continue to receive immediately released results through the portal.

**Table 1.** Survey Responses and Respondent Demographics

	No. (%)^a^				
	UC Davis Health	CU Anschutz	UTSW	VUMC	All sites
**Surveys**					
Total sent, No.	5221	15 000	11 048	12 522	43 791
Delivered	5189 (99.4)	14 856 (99.0)	10 929 (98.9)	12 406 (99.0)	43 380 (99.1)
Responses	1378 (26.6)	1656 (11.1)	2959 (27.1)	2146 (17.3)	8139 (18.8)
**Respondent characteristics**					
Gender					
Male	463 (33.6)	525 (31.7)	1132 (38.3)	775 (36.1)	2895 (35.6)
Female	913 (66.3)	1030 (62.2)	1816 (61.4)	1370 (63.8)	5129 (63.0)
Other or unknown	2 (<0.1)	101 (6.1)	11 (0.4)	1 (<0.1)	115 (1.4)
Age, median (IQR), y	63 (48-70)	64 (50-72)	67 (56-74)	64 (52-72)	64 (50-72)
Race^b^^,^^c^					
American Indian or Pacific Native	35 (2.5)	21 (1.3)	40 (1.4)	24 (1.2)	120 (1.5)
Asian	85 (6.2)	46 (2.8)	89 (3.0)	30 (1.4)	250 (3.1)
Black or African American	53 (3.8)	26 (1.6)	259 (8.8)	90 (4.3)	428 (5.3)
Native Hawaiian or Pacific Islander	11 (0.8)	3 (0.2)	7 (0.2)	2 (0.1)	23 (0.2)
White	1114 (80.8)	1440 (87.0)	2458 (83.1)	1888 (91.2)	6900 (84.8)
Other^d^	86 (6.2)	45 (2.7)	78 (2.6)	36 (1.7)	245 (3.0)
Spanish or Latino ethnicity	126 (9.1)	91 (5.5)	175 (5.9)	28 (1.3)	420 (5.2)
Language spoken at home^b^					
English	1277 (92.7)	1541 (93.1)	2852 (96.4)	2020 (94.1)	7690 (94.5)
Spanish	42 (3.0)	36 (2.2)	106 (3.6)	23 (1.1)	207 (2.5)
Chinese	9 (0.7)	5 (0.3)	15 (0.5)	5 (0.2)	34 (0.4)
Vietnamese	2 (0.1)	5 (0.3)	3 (0.1)	0	10 (0.1)
Korean	0	3 (0.2)	2 (0.1)	1 (<0.1)	6 (<0.1)
Russian	3 (0.2)	7 (0.4)	4 (0.1)	3 (0.1)	17 (0.2)
Arabic	4 (0.3)	0	7 (0.2)	2 (0.1)	13 (0.2)
Tagalog	6 (0.4)	1 (0.1)	8 (0.3)	4 (0.2)	19 (0.2)
Other	35 (2.5)	32 (1.9)	58 (2.0)	23 (1.1)	148 (1.8)
Comorbidities^b^					
Asthma or chronic lung disease	247 (17.9)	225 (13.6)	447 (15.1)	314 (14.6)	1255 (15.1)
Cancer	188 (13.6)	282 (17.0)	727 (24.6)	319 (14.9)	1516 (18.6)
Depression, anxiety, or other mental health problem	382 (27.7)	378 (22.8)	659 (22.3)	577 (26.9)	1996 (24.5)
Diabetes	175 (12.7)	183 (11.1)	488 (16.5)	388 (18.1)	1234 (15.2)
Hypertension	494 (35.8)	597 (36.1)	1423 (48.1)	990 (46.1)	3504 (43.1)
Heart disease	155 (11.2)	187 (11.3)	384 (13.0)	303 (14.1)	1029 (12.6)
Joint pain or arthritis	515 (37.4)	597 (36.1)	1091 (36.9)	829 (38.6)	3032 (37.3)
Stroke	48 (3.5)	47 (2.8)	111 (3.8)	71 (3.3)	277 (3.4)
Highest grade level or school completed					
Eighth grade or less	2 (0.1)	2 (0.1)	5 (0.2)	0	9 (0.1)
Some high school but did not graduate	7 (0.5)	12 (0.7)	13 (0.4)	4 (0.2)	36 (0.4)
High school graduate or GED	34 (2.5)	87 (5.3)	139 (4.7)	107 (5.0)	367 (4.5)
Some college, technical school, or 2-y degree	326 (23.7)	382 (23.1)	714 (24.1)	454 (21.2)	1876 (23.0)
4-y College graduate	341 (24.7)	389 (23.5)	767 (25.9)	527 (24.6)	2024 (24.9)
Some graduate school	114 (8.3)	128 (7.7)	273 (9.2)	153 (7.1)	668 (8.2)
Master’s or doctoral degree	482 (35.0)	565 (34.1)	990 (33.5)	790 (36.8)	2827 (34.7)
Current employment status					
Employed for wages	505 (36.6)	562 (33.9)	986 (33.3)	850 (39.6)	2903 (35.7)
Self-employed	69 (5.0)	106 (6.4)	187 (6.3)	134 (6.2)	496 (6.1)
Homemaker	24 (1.7)	55 (3.3)	67 (2.3)	65 (3.0)	211 (2.6)
Unemployed	23 (1.7)	27 (1.6)	27 (0.9)	20 (0.9)	97 (1.2)
Retired	600 (43.5)	735 (44.4)	1415 (47.8)	863 (40.2)	3613 (44.4)
Unable to work	72 (5.2)	60 (3.6)	175 (5.9)	82 (3.8)	389 (4.8)
Prefer not to answer	14 (1.0)	19 (1.1)	41 (2.1)	21 (1.0)	95 (1.2)
Work for health care organization					
Yes, in a clinical role	125 (9.1)	158 (9.5)	236 (8.0)	192 (8.9)	711 (8.7)
Yes, in a nonclinical role	109 (7.9)	118 (7.1)	243 (8.2)	258 (12.0)	728 (8.9)
No	1061 (77.0)	1282 (77.4)	2410 (81.4)	1563 (72.8)	6316 (77.6)

Abbreviations: CU Anschutz, University of Colorado Anschutz Medical Center; GED, general educational development; UC Davis Health, University of California, Davis Health; UTSW, University of Texas Southwestern Medical Center; VUMC, Vanderbilt University Medical Center.

^a^ All responses were self-reported.

^b^ Respondents could select multiple answers.

^c^ Categories for race are presented as the 6 options listed in the survey instrument.

^d^ “Other” was the option chosen on the survey.

**Table 2.** Patient Portal Preferences

	Completed responses, No./total No. (%)				
	UC Davis Health	CU Anschutz	UTSW	VUMC	All sites
Most frequent role for patient portal use in the past month					
Patient	1155/1309 (88.2)	1448/1570 (92.2)	2661/2936 (90.6)	1799/2053 (87.6)	7063/7868 (89.8)
Care partner	50/1309 (3.8)	47/1570 (3.0)	116/2936 (4.0)	102/2053 (5.0)	315/7868 (4.0)
Did not use patient portal	105/1309 (8.0)	75/1570 (4.8)	159/2936 (5.4)	152/2053 (7.4)	491/7868 (6.2)
Test type^a^					
Blood	767/999 (76.8)	1013/1358 (74.6)	1756/2332 (75.6)	1194/1587 (75.2)	4730/6276 (75.5)
COVID-19	201/999 (20.1)	187/1358 (13.8)	350/2332 (15.1)	170/1587 (10.7)	908/6276 (14.5)
Genetic	33/999 (3.3)	35/1358 (2.6)	72/2332 (3.1)	28/1587 (1.8)	168/6276 (2.7)
Imaging or biopsy	516/999 (51.7)	688/1358 (50.7)	1183/2332 (50.9)	657/1587 (41.4)	3044/6276 (48.6)
Other	169/999 (16.9)	165/1358 (12.2)	257/2332 (11.1)	255/1587 (16.1)	846/6276 (13.5)
Unknown	7/999 (0.7)	15/1358 (1.1)	26/2332 (1.1)	19/1587 (1.2)	67/6276 (1.1)
Test result					
Normal	471/999 (47.1)	734/1350 (54.4)	1381/2319 (59.6)	996/1578 (63.1)	3582/6246 (57.3)
Not normal	333/999 (33.3)	427/1350 (31.6)	597/2319 (25.7)	384/1578 (24.3)	1741/6246 (27.9)
Other	149/999 (14.9)	156/1350 (11.6)	258/2319 (11.1)	162/1578 (10.3)	725/6246 (11.6)
Unknown	46 (4.6)	33/1350 (2.4)	83/2319 (3.6)	36/1578 (2.3)	198/6246 (3.2)
Did a nurse or doctor explain the reason for the test before it was ordered?					
Yes	920/995 (92.5)	1242/1351 (91.9)	2115/2311 (91.5)	1490/1588 (93.8)	5767/6245 (92.3)
No	75/995 (7.5)	109/1351 (8.1)	196/2311 (8.5)	98/1588 (7.7)	478/6245 (7.7)
Where did you go for more information?^a^					
A family member or relative	82/883 (9.3)	125/1178 (10.6)	150/1940 (7.7)	122/1317 (9.3)	479/5318 (9.0)
A friend	30/883 (3.4)	34/1178 (2.9)	41/1940 (2.1)	24/1317 (1.8)	129/5318 (2.4)
Another health care practitioner	138/883 (15.6)	179/1178 (15.2)	260/1940 (13.4)	172/1317 (13.1)	749/5318 (14.1)
Someone I work with	10/883 (1.1)	14/1178 (1.2)	18/1940 (0.9)	13/1317 (1.0)	55/5318 (1.0)
Social media	5/883 (0.6)	7/1178 (0.6)	19/1940 (1.0)	8/1317 (0.6)	39/5318 (0.7)
An internet search	427/883 (48.4)	496/1178 (42.1)	741/1940 (38.2)	459/1317 (34.9)	2123/5318 (39.9)
Other	101/495 (11.4)	72/1178 (6.1)	112/1940 (5.8)	79/1317 (6.0)	364/5318 (6.8)
Did not seek additional information	307/883 (34.8)	467/1178 (39.6)	889/1940 (45.8)	640/1317 (48.6)	2303/5318 (43.3)
How were you contacted?^a^^,^^b^					
In-person or telemedicine visit	170/993 (17.1)	247/1339 (18.4)	530/2290 (23.1)	210/1578 (13.3)	1157/6200 (18.7)
Letter in the mail	19/993 (1.9)	34/1339 (2.5)	27/2290 (1.2)	43/1578 (2.7)	123/6200 (2.0)
Message through patient portal	607/993 (61.1)	771/1339 (57.6)	1348/2290 (58.9)	1057/1578 (67.0)	3783/6200 (61.0)
Telephone call	151/993 (15.2)	300/1339 (22.4)	367/2290 (16.0)	290/1578 (18.4)	1108/6200 (17.9)
Telephone voicemail	32/993 (3.2)	62/1339 (4.6)	68/2290 (3.0)	60/1578 (3.8)	222/6200 (3.6)
Other	49/993 (4.9)	76/1339 (5.7)	100/2290 (4.4)	68/1578 (4.3)	293/6200 (4.7)
I was never contacted	159/993 (16.0)	158/1339 (11.8)	304/2290 (13.3)	161/1578 (10.2)	782/6200 (12.6)
Future contact preference^a^					
Letter in the mail	22/1304 (1.7)	38/1561 (2.4)	56/2903 (1.9)	55/2046 (2.7)	171/7814 (2.2)
Telephone call	203/1304 (15.6)	306/1561 (19.6)	525/2903 (18.1)	411/2046 (20.1)	1445/7814 (18.5)
Text message	444/1304 (34.0)	348/1561 (22.3)	757/2903 (26.1)	542/2046 (26.5)	2091/7814 (26.8)
View on patient portal	1115/1304 (85.5)	1406/1561 (90.1)	2670/2903 (92.0)	1855/2046 (90.7)	7046/7814 (90.2)
Other	68/1304 (5.2)	60/1561 (3.8)	105/2903 (3.6)	67/2046 (3.1)	300/7814 (3.8)
Do not know	13/1304 (1.0)	15/1561 (1.0)	19/2903 (0.7)	17/2046 (0.8)	64/7814 (0.8)
Would like to continue receiving immediately released test results					
Yes	1231/1307 (94.2)	1514/1569 (96.5)	2803/2928 (95.7)	1972/2055 (96.0)	7520/7859 (95.7)
No	42/1307 (3.2)	35/1569 (2.2)	80/2928 (2.7)	44/2055 (2.1)	201/7859 (2.6)
Other	34/1307 (2.6)	20/1569 (1.3)	45/2928 (1.5)	39/2055 (1.9)	138/7859 (1.8)

Abbreviations: CU Anschutz, University of Colorado Anschutz Medical Center; UC Davis Health, University of California, Davis Health; UTSW, University of Texas Southwestern Medical Center; VUMC, Vanderbilt University Medical Center.

^a^ Respondents could select multiple answers.

^b^ Refers to communication following the release of a test result. This contact may have occurred either before or after the patient reviewed their result in the patient portal.

**Figure 1.**
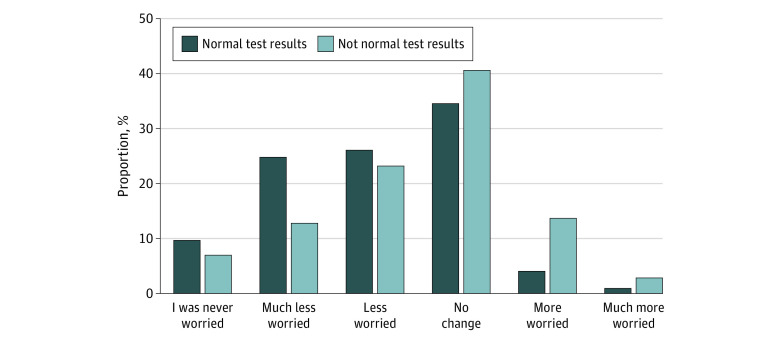
Percentage of Patients at Each Level of Worry, Stratified by Normal vs Not Normal Test Results

As shown in Figure 1, few respondents (411 of 5473 [7.5%]) reported being more worried after viewing test results. Among respondents who viewed a result before being contacted by a health care practitioner, almost half (2513 of 5473 [45.9%]) reported feeling less worried after reviewing their results through the patient portal. Among those reporting not normal results, most reported less or no change in their level of worry (2039 of 2442 [83.5%]). However, respondents who viewed not normal results were more likely to report being more worried or much more worried than those who reported normal results (403 of 2442 [16.5%] vs 294 of 5918 [5.0%]) (Figure 1). Among respondents with not normal blood test and imaging results, 187 of 1168 (16.0%) and 146 of 833 (17.5%), respectively, reported more worry or much more worry compared with those with normal blood test and imaging results (123 of 3078 [4.0%] and 104 of 1791 [5.8%], respectively).

All single-site adjusted models evaluating worry as a function of test result normality had significant overall model and partial effects, suggesting an association between not normal results and increased worry. The only other covariates associated with worry were other language at UC Davis Health (OR 0.30; 99% CI, 0.11-0.77) and precounseling at UC Davis Health (OR, 0.47; 99% CI, 0.24-0.92) and UTSW (OR, 0.64; 99% CI, 0.42-0.97).

**Figure 2.**
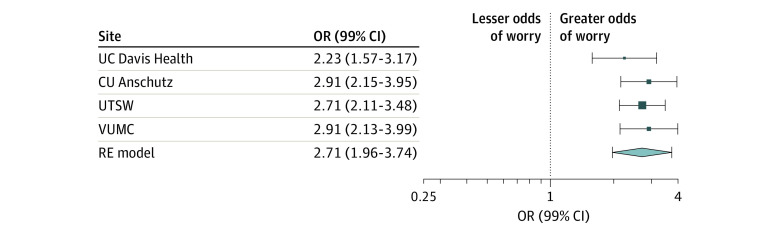
Adjusted Pooled Odds Ratios (ORs) Using a Random-Effects Model of Patient Worry as a Function of Whether a Test Result Was Not Normal Normal test result was the reference value. *I*^2^ for heterogeneity was less than 0.01%, suggesting very low intersite heterogeneity. Markers indicate ORs, with horizontal lines indicating 99% CIs; diamond indicates the pooled estimate, with outer points of the diamond indicating the 99% CI of the pooled estimate. CU Anschutz indicates University of Colorado Anschutz Medical Center; UC Davis Health, University of California, Davis Health; RE, random-effects; UTSW, University of Texas Southwestern Medical Center; and VUMC, Vanderbilt University Medical Center.

**Figure 3.**
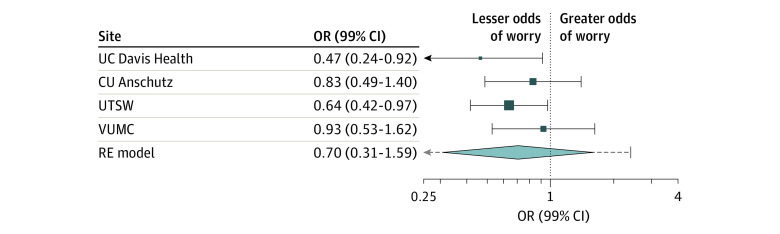
Adjusted Pooled Odds Ratios (ORs) Using a Random-Effects Model Evaluating the Association Between Precounseling Patients About the Reasons for Ordering a Test and Level of Worry *I*^2^ for heterogeneity was 36.50%, suggesting moderate intersite heterogeneity. Markers indicate ORs, with horizontal lines indicating 99% CIs; diamond indicates the pooled estimate, with outer points of the diamond indicating the 99% CI of the pooled estimate. CU Anschutz indicates University of Colorado Anschutz Medical Center; UC Davis Health, University of California, Davis Health; RE, random-effects; UTSW, University of Texas Southwestern Medical Center; and VUMC, Vanderbilt University Medical Center.

The pooled random-effects model evaluating worry as a function of test result normality indicated that not normal results were associated with greater likelihood of worry compared with normal results (pooled OR, 2.71; 99% CI, 1.96-3.74). Figure 2 shows individual and pooled adjusted ORs. The *I*^2^ statistic for the pooled model was 0.01%, suggesting very low heterogeneity. While site-specific models from 2 sites suggested that precounseling might be associated with less likelihood of worry, results of the pooled random-effects model evaluating worry as a function of precounseling were not significant (pooled OR, 0.70; 99% CI, 0.31-1.59). The *I*^2^ for this pooled model was 36.50%, suggesting moderate heterogeneity. Figure 3 shows individual and pooled adjusted ORs. Additional site-specific models including interaction terms between test result normality and precounseling showed that the interaction was not significant.

## Discussion

We surveyed a large cohort of patients and care partners at 4 geographically distributed academic medical centers who had accessed the patient portal at least once in the past year. Nearly all respondents (95.7%) wanted to continue to receive test results through the online patient portal immediately upon reporting and before being contacted by a health care practitioner. Most respondents indicated that reviewing results had either a positive effect or no effect on their level of worry. However, a subset of respondents with not normal results experienced additional worry. At 2 institutions, we observed reduced worry associated with precounseling before testing.

Few prior studies have investigated patient attitudes and preferences related to open results. Early work by Giardina et al^5,37^ found an association between receiving abnormal results and negative emotions, highlighting a need for more nuanced and customizable result release strategies. During the studies by Giardina et al,^5,37^ available test results were commonly released at tiered intervals through the patient portal based on sensitivity and perceived risk of misinterpretation.^13^ Since the Cures Act Final Rule, multiple studies have highlighted the risk of worry, the need to improve result interpretation by patients, and the need for medical counseling.^18,23,38,39^ However, these studies were conducted with small cohorts at single sites.

The open notes literature has highlighted the importance of data availability and transparency to enable patients to manage their health care.^11,16,20,40,41^ Our findings suggest that open results may have a similar effect, as most respondents sought additional information after viewing results. Interestingly, 39.9% of patients who sought additional information after reviewing their results conducted an internet search, highlighting potential unmet information needs. Providing patients time to review, research, and process their own test results might allow them to prepare for subsequent discussions with their health care practitioners and may lead to better shared decision-making.

A subset of respondents reported additional worry after viewing not normal results. Our modeling results support the findings reported by Giardina and colleagues^5^ that patients receiving not normal results are at increased risk for negative emotions, potentially due to difficulty interpreting the results in the context of their own health. Prior literature^42,43^ has highlighted a similar trend in worry when receiving news of abnormal results outside a patient portal, such as through a telephone call or during an in-person visit. We found that 95.3% of participants who received abnormal test results would like to continue to receive immediately released results through the portal. This finding suggests that there may be benefits to receiving abnormal results online, such as allowing patients to choose where and with whom to view such results. Additional research is necessary to better understand the nuance of worry from receiving abnormal test results, especially as it relates to release through the portal. A separate qualitative evaluation of the free-text questions in our survey is forthcoming and may provide insight into this phenomenon.

A large proportion of respondents (92.3%) reported receiving precounseling. Interestingly, we found no association between precounseling and lower levels of worry. Best practices for precounseling should be studied further. Additionally, the workflow and financial consequences of this added task for an already stressed clinical workforce warrants further consideration. Precounseling strategies might encompass both technical and social-technical approaches, including in-person anticipatory guidance, improved asynchronous communication, and portal-based educational materials. Other strategies include optimizing existing patient portal interfaces to give users control over their notification preferences related to sensitive or abnormal results or timing the release of test results during working hours. Additional research is necessary to further investigate the efficacy of strategies to mitigate emotional distress.

### Limitations

This study has limitations. Patient portal users were surveyed at 4 large academic medical centers that were geographically distributed across the US. Results may not be generalizable to patients outside these systems. Further, all sites used Epic’s MyChart patient portal. It is possible that vendor differences could influence user perceptions, though portal functionality is similar between most vendors. Our study relied on self-reported responses, which may introduce biased or potentially incorrect responses. The response rate was modest, with variation between sites. It is possible respondents were more enthusiastic about open results and do not represent all portal users. Similarly, only patients who accessed test results via the patient portal were included in the study cohort, which may bias our findings. Three of 4 sites used research registries for recruitment, which may have contributed to heterogeneous response rates between sites. However, the large sample size reinforces our findings and enabled robust statistical testing among subsets of respondents.

A survey question about sex was erroneously omitted from 3 of the sites (UC Davis Health, UTSW, and VUMC) but was included at CU Anschutz. We obtained aggregate sex data from the EHR for the respondent cohort for these 3 sites but were unable to link them at a participant level. Therefore, sex was not included in the multivariable models. This also explains the higher missingness in sex data reported at CU Anschutz.

Survey respondents were primarily White, female, English-speaking, and highly educated. Prior studies indicated a similar demographic profile among patient portal users, suggesting a bias in self-selecting portal users.^44^ Future studies should capture preferences among non–English-speaking patients and patients from underrepresented racial and ethnic populations and with underrepresented educational levels and socioeconomic status.

## Conclusions

This survey study assessed attitudes and perceptions related to immediately released test results in a large cohort of patients and caregivers at 4 geographically distributed academic medical centers. Most respondents preferred to receive test results through the patient portal even if it meant viewing results prior to discussion with a health care professional. This remained true for patients receiving not normal results. However, receiving a result that was not normal was associated with increased worry compared with receiving a normal result. As health care systems continue to navigate this new era of health information transparency, balancing patients’ expectation of immediate access to their information with the need to manage increased worry and health care practitioner burden is increasingly important.

## Notes

Supplement 1. **eAppendix.** Patient Attitudes About Immediate Access to Test Results **eTable.** Comparison of Demographic Characteristics, Experiences, and Preferences Among Respondents Who Received Precounseling vs Those Who Did Not

Supplement 2.
Data Sharing Statement
