# Supplementary material for: Perspectives of Patients About Immediate Access to Test Results Through an Online Patient Portal
Source: JAMA Netw Open. 2023 Mar 20;6(3):e233572. doi: 10.1001/jamanetworkopen.2023.3572 (PMC10028486; doi:10.1001/jamanetworkopen.2023.3572)
Supplement: Supplement 1. — eAppendix. Patient Attitudes About Immediate Access to Test Results eTable. Comparison of Demographic Characteristics, Experiences, and Preferences Among Respondents Who Received Precounseling vs Those Who Did Not [file jamanetwopen-e233572-s001.pdf]

Supplementary Online Content

Steitz BD, Turer RW, Lin CT, et al. Perspectives of patients about immediate access to test results through an online patient portal. *JAMA Netw Open*. 2023;6(3):e233572. doi:10.1001/jamanetworkopen.2023.3572

eAppendix. Patient Attitudes About Immediate Access to Test Results
eTable. Comparison of Demographic Characteristics, Experiences, and Preferences Among Respondents Who Received Precounseling vs Those Who Did Not

This supplementary material has been provided by the authors to give readers additional information about their work.

© 2023 Steitz BD et al. *JAMA Network Open*.

eAppendix

Patient Attitudes About Immediate Access to Test Results

Because of a new federal law, Vanderbilt Health has expanded the information available to you about your health in My Health at Vanderbilt, our online patient portal. This means you can see more of your test results and clinical notes than before, and can see them immediately after they are available. We are interested in your thoughts and invite you to take this survey, which should only take 5-10 minutes to complete.

During the past year, you may have received results of medical tests at Vanderbilt. Tests may have included blood work, COVID-19 results, pathology, and results from X-rays and MRIs.

In the past month, did you more often use My Health at Vanderbilt as a:

- ○ Patient
- ○ Care partner of a patient on My Health at Vanderbilt
- ○ Did not use My Health at Vanderbilt in the past month

Have you viewed results from your medical tests on My Health at Vanderbilt in the past month?

- ○ Yes
- ○ No

Which of the following best describes what you USUALLY do when waiting for test results?

- ○ I wait for my doctor's office to call me with the results
- ○ I wait for a notification from My Health at Vanderbilt
- ○ I check for the results in My Health at Vanderbilt before receiving a notification
- ○ Other (please specify)

Please specify

____

When you answer these questions, please think about the last test result you viewed on My Health at Vanderbilt. If you had results from several tests available on the same day, please think about the one that was most concerning to you.

Did a nurse or doctor explain why the test was being done before it was done?

- ○ Yes
- ○ No

What types of tests did you receive? Select all that apply.

- ☐ Blood test
- ☐ Imaging test like an x-ray, CAT scan, MRI, or ultrasound Biopsy
- ☐ Genetic test
- ☐ COVID-19 test
- ☐ Other (please specify)
- ☐ I don't know

Please specify

____

Why did your doctor or nurse order this test?

- ○ To look into new symptoms or new health problems you were having
- ○ To follow a health condition you know that you have
- ○ For regular screening (for example, checking your cholesterol)
- ○ Other (please specify)

© 2023 Steitz BD et al. *JAMA Network Open*.

Please specify	__________________
What was the result?	◯ Normal<br>◯ Not Normal<br>◯ Other (please specify)<br>◯ I don't know
Please specify	__________________
Did you look at this test result on My Health at Vanderbilt before you were contacted by a doctor or nurse about it?	◯ Yes<br>◯ No<br>◯ I don't know
Did looking at the results of your medical test before you could discuss it with a clinician (doctor or nurse) make you more or less worried about your health?	◯ Much less worried<br>◯ Less worried<br>◯ No change<br>◯ More worried<br>◯ Much more worried<br>◯ I was never worried
Has something happened (good or bad) as a result of seeing your medical test results before you could discuss it with a doctor or nurse? If so, please explain:	__________________
Where did you go for more information about the results of your medical tests? Select all that apply.	☐ A family member or relative<br>☐ A friend<br>☐ Another health care provider like a doctor or nurse<br>☐ Someone I work with<br>☐ Social media like Facebook, Twitter, or Instagram<br>☐ An Internet search (please specify)<br>☐ Other (please specify)<br>☐ I did not seek additional information
Please specify	__________________
How confusing was viewing this test result on My Health at Vanderbilt?	◯ Very confusing<br>◯ Somewhat confusing<br>◯ Not very confusing<br>◯ Not confusing at all<br>◯ Not sure
What was confusing about viewing your medical tests on My Health at Vanderbilt? Select all that apply.	☐ The test result was difficult to find<br>☐ I did not know how to interpret the test<br>☐ I had never used My Health at Vanderbilt before<br>☐ The result was for a test that I did not know I had<br>☐ Other (please specify)
Please specify	__________________

© 2023 Steitz BD et al. *JAMA Network Open*.

How did the doctor or nurse contact you about your results? Select all that apply.	☐ Sent a message through My Health at Vanderbilt<br>☐ Spoke with me over the phone<br>☐ Left a voice message on the phone<br>☐ At an in-person or telemedicine visit<br>☐ Sent a letter through the mail<br>☐ Other (please specify)<br>☐ I was never contacted by a doctor or nurse
Please specify	__________________________________
In the future, how would you like to receive new test results? Select all that apply.	☐ Letter in the mail<br>☐ Phone call from a healthcare professional<br>☐ Text message from Vanderbilt Health<br>☐ View it on My Health at Vanderbilt<br>☐ Other (please specify)<br>☐ Don't know/not sure
Please specify	__________________________________
In the future, would you like to be notified that you have a new test result on My Health at Vanderbilt even if it means that you may see a result before your doctor or nurse is able to contact you about the result?	◯ Yes<br>◯ No<br>◯ Other (Please specify)
Please specify	__________________________________
Is there anything else you'd like to share regarding your experience with checking test results through My Health at Vanderbilt?	__________________________________

© 2023 Steitz BD et al. *JAMA Network Open*.

Demographics

In general, how would you rate your health?	◯ Excellent<br>◯ Very good<br>◯ Good<br>◯ Fair<br>◯ Poor
Have you been told by a doctor that you have any of the following? Select all that apply.	☐ Hypertension or high blood pressure<br>☐ Heart disease, including heart attack<br>☐ Diabetes<br>☐ Asthma or chronic lung disease such as chronic bronchitis, emphysema or COPD<br>☐ Depression, anxiety or other mental health problems<br>☐ Cancer<br>☐ Joint pain or arthritis<br>☐ Had a stroke
What is the highest grade or level of school that you have completed?	◯ 8th grade or less<br>◯ Some high school, but did not graduate<br>◯ High school graduate or GED<br>◯ Some college, technical school, or 2-year degree<br>◯ 4-year college graduate<br>◯ Some graduate school<br>◯ Masters or Doctoral degree
Are you of Spanish/Hispanic/Latino ethnicity?	◯ Yes<br>◯ No
What do you consider to be your racial background? Check all that apply.	☐ American Indian or Pacific Native<br>☐ Asian<br>☐ Black or African American<br>☐ Native Hawaiian or Pacific Islander<br>☐ White<br>☐ Other
What language(s) do you usually speak at home? Check all that apply.	☐ English<br>☐ Spanish<br>☐ Chinese<br>☐ Vietnamese<br>☐ Korean<br>☐ Russian<br>☐ Arabic<br>☐ Tagalog<br>☐ Other
Please specify	________________________________
Which of the following best describes your current employment status?	◯ Employed for wages<br>◯ Self-employed<br>◯ Homemaker<br>◯ Unemployed<br>◯ Retired<br>◯ Unable to work<br>◯ Prefer not to answer
Do you work in a health care organization?	◯ Yes, in a clinical role<br>◯ Yes, in a non-clinical role<br>◯ No

© 2023 Steitz BD et al. *JAMA Network Open*.

What is your gender?	◯ Female<br>◯ Male<br>◯ Other<br>◯ Prefer not to answer
What is your age?	________________
In what zip code is your home address?	________________

© 2023 Steitz BD et al. *JAMA Network Open*.

In what state is your home address?	◯ Alabama<br>◯ Alaska<br>◯ Arizona<br>◯ Arkansas<br>◯ California<br>◯ Colorado<br>◯ Connecticut<br>◯ Delaware<br>◯ Florida<br>◯ Georgia<br>◯ Hawaii<br>◯ Idaho<br>◯ Illinois<br>◯ Indiana<br>◯ Iowa<br>◯ Kansas<br>◯ Kentucky<br>◯ Louisiana<br>◯ Maine<br>◯ Maryland<br>◯ Massachusetts<br>◯ Michigan<br>◯ Minnesota<br>◯ Mississippi<br>◯ Missouri<br>◯ Montana<br>◯ Nebraska<br>◯ Nevada<br>◯ New Hampshire<br>◯ New Jersey<br>◯ New Mexico<br>◯ New York<br>◯ North Carolina<br>◯ North Dakota<br>◯ Ohio<br>◯ Oklahoma<br>◯ Oregon<br>◯ Pennsylvania<br>◯ Rhode Island<br>◯ South Carolina<br>◯ South Dakota<br>◯ Tennessee<br>◯ Texas<br>◯ Utah<br>◯ Vermont<br>◯ Virginia<br>◯ Washington<br>◯ West Virginia<br>◯ Wisconsin<br>◯ Wyoming

© 2023 Steitz BD et al. *JAMA Network Open*.

eTable. Comparison of Demographic Characteristics, Experiences, and Preferences Among Respondents Who Received Precounseling vs Those Who Did Not

	Responses, No. (%)		
	**Received Pre-Counseling**	**Did Not Receive Pre-Counseling**	**All Responses**
	(n=5767)	(n=478)	(n=6245)
Race (Multiple Select)			
American Indian or Pacific Native	90 (1.6)	8 (1.7)	98 (1.6)
Asian	166 (2.9)	18 (3.8)	184 (2.9)
Black or African American	330 (5.7)	21 (4.4)	351 (5.6)
Native Hawaiian or Pacific Islander	17 (0.3)	1 (0.2)	18 (0.3)
White	5038 (87.4)	405 (84.7)	5443 (87.2)
Other	182 (3.2)	23 (4.8)	205 (3.3)
Ethnicity			
Spanish or Latino	305 (5.3)	29 (6.1)	334 (5.8)
Languages spoken at home (Multiple Select)			
English	5618 (97.4)	457 (95.6)	6075 (97.3)
Spanish	154 (2.7)	16 (3.3)	170 (2.7)
Chinese	21 (0.4)	1 (0.2)	22 (0.4)
Vietnamese	6 (0.1)	1 (0.2)	7 (0.1)
Korean	4 (0.1)	1 (0.2)	5 (0.1)
Russian	12 (0.2)	1 (0.2)	13 (0.2)
Arabic	10 (0.2)	3 (0.6)	13 (0.2)
Tagalog	15 (0.3)	1 (0.2)	16 (0.3)
Other	101 (1.8)	15 (3.1)	116 (1.9)
Comorbidities (Multiple Select)			
Asthma or chronic lung disease	933 (16.2)	95 (19.9)	1028 (16.5)
Cancer	1152 (20.0)	100 (20.9)	1252 (20.0)
Depression, anxiety, or other mental health problem	1478 (25.6)	123 (25.7)	1601 (25.6)
Diabetes	973 (16.9)	85 (17.8)	1058 (16.9)
Hypertension	2648 (45.9)	219 (45.8)	2867 (45.9)
Heart disease	818 (14.2)	63 (13.2)	881 (14.1)
Joint pain or arthritis	2297 (39.8)	176 (36.8)	2473 (39.6)
Stroke	205 (3.6)	25 (5.2)	230 (3.7)
Highest grade level or school completed			
8th grade or less	6 (0.1)	2 (0.4)	8 (0.1)
Some high school but did not graduate	23 (0.4)	3 (0.6)	26 (0.4)
High school graduate or GED	265 (4.7)	35 (7.5)	300 (4.9)
Some college, technical school, or two-year degree	1399 (24.6)	103 (22.0)	1502 (24.4)
Four-year college graduate	1458 (25.6)	111 (23.7)	1569 (25.4)
Some graduate school	493 (8.7)	53 (11.3)	546 (8.9)
Masters or Doctoral degree	2053 (36.0)	162 (34.5)	2215 (35.9)
Current Employment Status			
Employed for wages	2088 (36.6)	145 (31.0)	2233 (36.2)
Self-employed	359 (6.3)	41 (8.8)	400 (6.5)
Homemaker	156 (2.7)	10 (2.1)	166 (2.7)
Unemployed	57 (1.0)	10 (2.1)	67 (1.1)
Retired	2679 (47.0)	218 (46.6)	2897 (46.9)
Unable to work	294 (5.2)	36 (7.7)	330 (5.3)
Prefer not to answer	70 (1.2)	8 (1.7)	78 (1.3)
Work for healthcare organization			
Yes, in a clinical role	524 (9.2)	42 (9.1)	566 (9.2)
Yes, in a non-clinical role	527 (9.3)	28 (6.0)	555 (9.1)
No	4614 (81.4)	394 (84.9)	5008 (81.7)
Result			
Normal	3310 (57.8)	243 (51.5)	3553 (57.4)
Not Normal	1603 (28.0)	126 (26.7)	1729 (27.9)
Other	648 (11.3)	68 (14.4)	716 (11.6)
Unknown	161 (2.8)	35 (7.4)	196 (3.2)

© 2023 Steitz BD et al. *JAMA Network Open*.
