# Supplementary material for: Perspectives of Patients About Immediate Access to Test Results Through an Online Patient Portal
Source: JAMA Netw Open. 2023 Mar 20;6(3):e233572. doi: 10.1001/jamanetworkopen.2023.3572 (PMC10028486; doi:10.1001/jamanetworkopen.2023.3572)
Supplement: Supplement 2. — Data Sharing Statement [file jamanetwopen-e233572-s002.pdf]

Data Sharing Statement

Steitz. Perspectives of Patients About Immediate Access to Test Results Through an Online Patient Portal. *JAMA Netw Open*. Published March 20, 2023.
doi:10.1001/jamanetworkopen.2023.3572

Data
Data available: No

Additional Information
Explanation for why data not available: Our dataset contain patient-level responses, which our IRB has deemed unacceptable to share.
